# Does treatment of subsyndromal depression improve depression and diabetes related outcomes: protocol for a randomised controlled comparison of psycho-education, physical exercise and treatment as usual

**DOI:** 10.1186/1745-6215-12-17

**Published:** 2011-01-21

**Authors:** Mirjana Pibernik-Okanović, Dea Ajduković, Marijana Vučić Lovrenčić, Norbert Hermanns

**Affiliations:** 1 Team for Mental Health, Vuk Vrhovac University Clinic for Diabetes, Endocrinology and Metabolic Diseases, Merkur Teaching Hospital, University of Zagreb, Zagreb, Croatia; 2 Institute of Clinical Chemistry and Laboratory Medicine, Merkur Teaching Hospital, University of Zagreb, Zagreb, Croatia; 3 Research Institute of the Diabetes Academy Mergentheim, Bad Mergentheim, Germany

## Abstract

**Background:**

The prevalence of mood difficulties in persons with diabetes is approximately twice that in the general population, affecting the health outcomes and patients' quality of life in an undesirable way. Although subsyndromal depression is an important predictor of a more serious clinical depression, it is often overlooked. This study aims to compare the effects of two non-pharmacological interventions for subsyndromal depression, psychoeducation and physical exercise, with diabetes treatment as usual on mood- and diabetes-related outcomes.

**Methods and Design:**

Type 2 diabetic patients aged 18-65 yrs. who report mood difficulties and the related need for help in a mail survey will be potential participants. After giving informed consent, they will be randomly assigned to one of the three groups (psychoeducation, physical activity, treatment as usual). Depressive symptoms, diabetes distress, health-related quality of life and diabetes self-care activities will be assessed at baseline, at 6 weeks, 6 months and 12 months. A structured clinical interview for DSM-IV Axis I Disorders (SCID-I) will be performed at baseline and at one-year follow-up in order to determine the clinical significance of the patients' depressive symptoms. Disease-related data will be collected from patients' files and from additional physical examinations and laboratory tests.

The two interventions will be comparable in terms of format (small group work), duration (six sessions) and approach (interactive learning; supporting the participants' active roles). The group treated as usual will be informed about their screening results and about the importance of treating depression. They will be provided with brief re-education on diabetes and written self-help instructions to cope with mood difficulties.

Primary outcomes will be depressive symptoms. Secondary outcomes will be glycaemic control, diabetes-related distress, self-management of diabetes and health-related quality of life. Tertiary outcomes will be biochemical markers reflecting common pathophysiological processes of insulin resistance, inflammation and oxidative damage that are assumed to be intertwined in both diabetes and depression. The mixed-effect linear model will be used to compare the outcome variables.

Power analysis has indicated that the two intervention groups and the control group should comprise 59 patients to enable detection of clinically meaningful differences in depressive symptoms with a power of 80% and alpha = 0.05. Outcomes will be analysed on an intention-to-treat basis.

**Trial Registration:**

ISRCTN: ISRCTN05673017

## Background

The prevalence of clinical depression in diabetic patients is approximately twice that in the general population [1]. The literature data have consistently demonstrated a synergistic interaction between diabetes and depression that increases the risk for poor health outcomes [2]. Compared to patients with diabetes alone, patients with depression and diabetes have poorer self-management (i.e. adherence to diet, exercise regimen and blood glucose monitoring) and significantly more lapses in refilling oral hypoglycaemic, lipid-lowering and antihypertensive prescriptions [3,4]. They are also significantly more likely to have cardiac risk factors such as smoking, obesity and sedentary lifestyle [5]. Depression is associated with an increased risk of metabolic dysregulation [6], micro- and macrovascular complications [7], and mortality [8]. However, despite the clear evidence of an undesirable interaction between depression and diabetes, depression remains unrecognised in approximately one half of diabetic patients, and is consequently not treated properly [9].

Both psychiatric medication [10-12] and psychological interventions [13-16] have been proven to be efficient in alleviating depression in diabetic patients. However, the effects of pharmacological treatment of depression on glycaemic control remain unclear, with some trials pointing to adverse effects, and some reporting on modest positive effects on HbA_1c _values. Trials relying on cognitive behavioural therapy and counselling for depression have shown moderate to good effects on glycaemic control [13-15]. An exception is the study by Georgiades et al. [17], which demonstrated that changes in depressive symptoms after a cognitive behavioural therapy intervention were not associated with changes in HbA_1c _values over a one-year period.

Recent research has suggested that subsyndromal depression, defined as the presence of depressive symptoms that fall short of full diagnostic criteria for major depression or dysthymia, has a profound influence on the affected patients' quality of life and may be a part of a continuum of depressive disorders [18,19]. Data from the general population indicate that spontaneous remission rates for this type of mood disturbance are low [20]. Subsyndromal depression was found to increase the risk of subsequent major depression [21] and suicide [22]. Chronic illness and medical burden are among the predictors of conversion from minor depression into its more severe clinical forms [23,24]. Treating depression in diabetic patients at its early stages may therefore be beneficial for improving both the affected patients' mood and the self-management of diabetes.

Studies exploring the effects of treatment for mild to moderate depression in diabetic patients are scarce, although this form of depression is highly prevalent in the diabetic population [1]. However, both pharmacological treatment [25] and psychoeducation [26,27] appear to be promising as treatment for mild depression and for improving glycaemic control in these patients. Physical activity has been demonstrated to alleviate depressive symptoms in the non-diabetic population as efficiently as cognitive behavioural therapy [28]. Lack of exercise in diabetic patients is associated with 72% to 75% higher likelihood of being depressed [29]. Two randomised trials have examined the impact of a depression-specific intervention on exercise patterns in persons with type 2 diabetes. They have provided equivocal support for the impact, one suggesting an increase in physical activity [15] and the other reporting no such change [30].

Research on the treatment of subsyndromal depression in patients with diabetes is scant, allowing no conclusion about its effects on depression- and diabetes-related outcomes. Due to a small number of studies in the field, it remains unclear whether some non-pharmacological therapeutic approaches may be superior to others.

There is an increasing body of evidence implying chronic inflammation as an important link between diabetes and depression [31]. Activation of innate immunity and complex changes in inflammatory patterns have been found to play a significant role in the pathogenesis of type 2 diabetes and insulin resistance [32], and a recent hypothesis on the pivotal role of inflammatory cytokines and oxidative/nitrosative stress in the pathogenesis of depression has recieved much attention [33]. Whether or not inflammatory response and the resulting oxidative damage in diabetes is primary or reactive is not clear, but its significant association with both insulin resistance and beta cell dysfunction, as well as with many different co-morbidities (atherosclerosis, obesity, depression), definitively suggests that the activation of innate immunity should be regarded as more than just an epiphenomenon.

Despite an accumulating body of evidence suggesting that changes in inflammatory and pro-/anti-oxidative biomarkers in diabetes and clinical depression are separate entities, little is known on the possible involvement of these complex processes in diabetic patients with subsyndromal depression. Our study aims to investigate whether non-pharmacological intervention for subsyndromal depression, apart from the hypothesised alleviation of depressive symptoms and improvement in glycaemic control, could offer additional benefit to diabetic patients by reducing chronic inflammation and oxidative damage.

## Methods and Design

### Objective

The aim of this paper is to present a research protocol of a randomised controlled trial designed to compare two non-pharmacological interventions for subsyndromal depression (psychoeducation and physical activity) with diabetes treatment as usual. We hypothesise that the two interventions will be superior to treatment as usual in reducing depressive symptoms and improving diabetes control.

### Design

**Figure 1:**
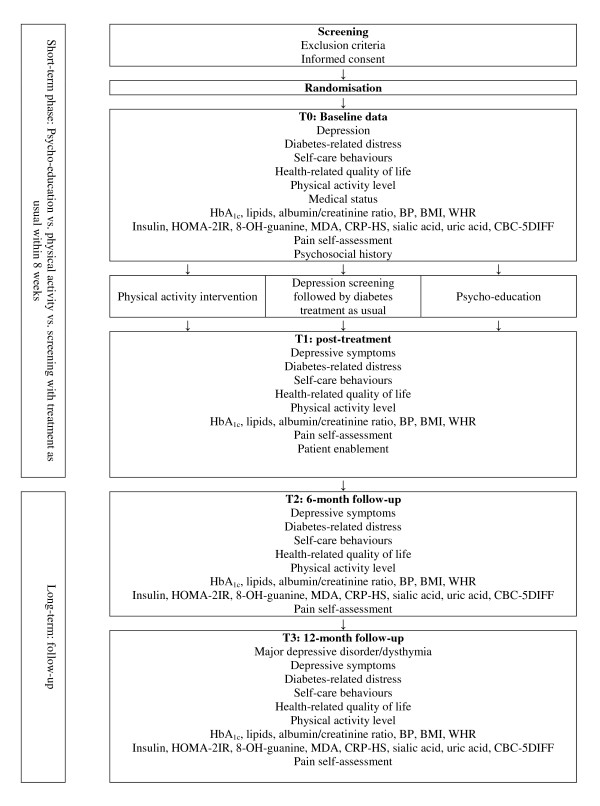
**Study diagram**.

Type 2 diabetic patients will be randomised to a six-week psycho-educational intervention, a six-week physical activity intervention, or diabetes treatment as usual. The study diagram is presented in Figure 1.

### Setting

The study will be conducted in a specialised diabetes clinic (The Vuk Vrhovac University Clinic for Diabetes, Endocrinology and Metabolic Diseases) that is a part of a teaching hospital (Merkur Teaching Hospital) affiliated with the University of Zagreb in Croatia.

### Inclusion criteria

The eligible participants have to meet all of the following criteria:

• type 2 diabetic patients

• aged 18-65 yrs.

• attended a regular check-up in the Clinic during the previous year

• living in Zagreb

• self-reported mood difficulties on PHQ-2 in a mail survey

• expressed the need for additional help regarding mood difficulties

### Exclusion criteria

• poor literacy and/or lack of fluency in Croatian language

• mobility difficulties

• visual impairment

• current major depressive disorder

• current or history of alcohol abuse, bipolar or psychotic disorder

• medical contraindications for physical exercise (heart attack or stroke during the past 12 months, angina pectoris)

### Recruitment and baseline

A standardised two-item screening instrument, the Patient Health Questionnaire (PHQ-2) that includes an additional question inquiring into patients' need to receive help in mood-related issues [34] will be sent to all potential study participants retrieved from a database of diabetic patients [35]. Participants will be provided with a reply-paid envelope for the return of the completed questionnaire. They will also be asked for permission to be contacted by telephone if they indicate that they are interested in receiving help.

Participants who return the questionnaire reporting mood difficulties and the need for help will be telephoned for the assessment of exclusion criteria. Those whose status of contraindications for exercise is unclear will be offered a medical examination by the Clinic's internist, who will determine whether this exclusion criterion is met. The potential participants will be interviewed for a clinical diagnosis of depression. SCID-I (Structured Clinical Interview for the DSM-IV Axis I Disorders) will be administered by a trained researcher by telephone, as this method has proved to be comparable to the face-to-face interview [36]. Patients who meet the criteria for the diagnosis of major depression will be advised to refer to their GP or psychiatrist and will be excluded from the trial, since they require more intensive treatment. The eligible patients will be offered an appointment with the researchers to give informed consent, provide data and be informed of the group assignment.

### Randomisation

This study design has three arms: psycho-educational intervention, physical activity intervention, and depression screening followed by diabetes treatment as usual. Eligible participants will be randomised into one of the three groups by using a computer-assisted approach. Groups will be balanced by age and sex. Randomisation will be carried out by a member of the team who will not take part in the treatment. Data on patients (SCID-I assessment, baseline and follow-up data) will be collected by team members unaware of the patients' group assignment. Baseline and one-year follow-up structured clinical interview (SCID-I) will be administered by the same person on both occasions.

### Interventions

#### Psychoeducation

The intervention will comprise 6 interactive small-group meetings (4-6 members), each lasting for 60-90 minutes. The topics will include: interaction of depression and diabetes; alleviating burden of depression through activities and problem solving; associations between depression and cognitive processes - thoughts, beliefs and attitudes that induce and maintain depression; gaining social support and developing a personal plan for managing problems in the future.

Meetings will be held at weekly intervals. The sessions will combine a short standardised presentation focused on particular topics, group discussions and homework assignments. Patients will be provided with a self-help manual for overcoming depressive difficulties based on the "Coping with depression" course by P.M. Lewinsohn. The manual is structured in a way that facilitates introducing personal examples and making notes. The "Coping with depression" programme is well-evaluated in both general population and medical patients [37]. For the purpose of this study, the programme has been adjusted to address specific emotional problems related to diabetes and adapted for a shorter format of this intervention. Patients will also receive a workbook containing exercises to recognise depressive symptoms, become aware of daily activity patterns, acquire problem-solving techniques, and recognize and modify cognitive patterns that contribute to the maintenance of depression. The manual was tested for comprehensibility and clarity in a group of diabetic patients (N = 8) with different demographic and disease-related characteristics.

#### Physical activity

The intervention will comprise 6 weekly small-group sessions. The purpose of these sessions will be to educate patients about the interaction of physical activity, mood and diabetes, and to increase their participation in a variety of physical activities with an emphasis on walking. The sessions will combine interactive lectures and exercise techniques (warm-up, flexibility, strengthening and stretching exercises) that are considered suitable for the study participants. The educational topics will include effects of exercise on mood; short- and long-term effects of exercise on blood glucose and the cardiovascular system; and strategies to develop and maintain a personal plan for regular exercise. The programme has been developed by a professional trainer experienced in working with the diabetic population.

The sessions will be organised in a group format and led by a professional trainer. Exercise intensity will be measured by a heart rate monitor and maintained in a light-to-medium intensity range. The volume of physical activity will be monitored by a pedometer for one week preceding each point of measurement.

#### Depression screening followed by diabetes treatment as usual

The patients screened for depression will be given an explanation of their results and informed about the importance of treating depression, as well as about different treatment options. They will be provided with brief re-education on diabetes aimed at alleviating diabetes-related burden, and with written self-help instructions to cope with mood difficulties.

Follow-up data will be collected at the same points of measurement as in the treatment groups.

### Assessments

**Figure 2:**
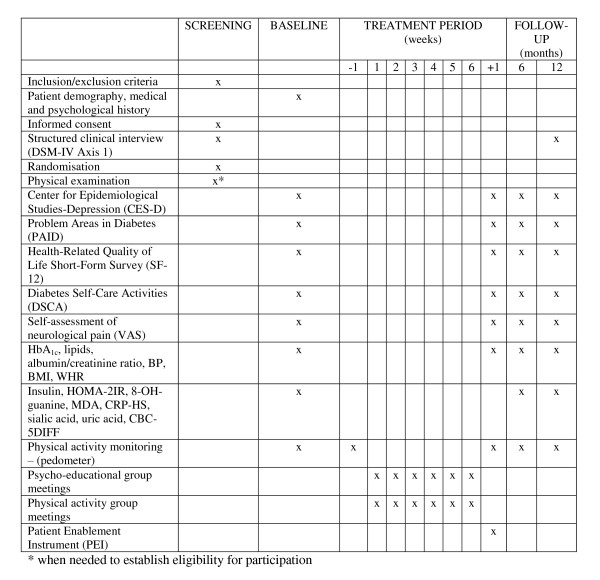
**Study flowchart**.

Patients will be assessed at four time points: at baseline (T0), at the end of the interventions (i.e., 6 weeks after the beginning, T1), at 6 months (T2) and at 12 months (T3) after the beginning of the intervention (Figure 2). Data will be collected by experienced researchers blinded to group assignment.

Data concerning the patients' psychological history (psychological morbidity, treatment method, symptom course, family history of psychiatric morbidity) and current psychosocial situation (family and professional status, economic circumstances, recent stress experiences, perceived social support) will be collected at baseline by semi-structured telephone interview.

Psychological questionnaires will be applied at all points of measurement to collect data on patients' emotional state and their experience in living with diabetes: depressive symptoms (Center for Epidemiologic Studies Depression Scale, CES-D), diabetes distress (Problem Areas in Diabetes, PAID), health-related quality of life (Short Form Survey, SF-12) and diabetes self-care (Summary of Diabetes Self-Care Activities, SDSCA). The questionnaires have been psychometrically evaluated in Croatian diabetic patients. SCID-I will be re-administered after 12 months.

To assess the level of physical activity, a subscale of the Summary of Diabetes Self-Care Activities (SDSCA) referring to the frequency and intensity of physical activity during previous week will be applied at all measurement points. To provide objective data, patients from both the intervention groups and the control group will wear the pedometer for one week preceding all points of measurement, including the baseline.

Baseline information on the type, duration and treatment of diabetes, body mass index (BMI), waist-to-hip ratio (WHR), and concomitant somatic diseases will be retrieved from the patients' medical records. If necessary, an internist will be consulted about the interpretation of data. Biochemical parameters including HbA_1c_, lipids, and albumin/creatinine ratio, and data on patients' body weight, blood pressure and diabetes medication will be determined and recorded at all points of measurement. Fasting plasma glucose and insulin will be used to assess insulin resistance by a HOMA-2IR model [38]. Low-grade inflammation will be monitored by high sensitivity C-reactive protein, total sialic acid [39], WBC and neutrophil counts. Urinary 8-hydroxy-2'-deoxyguanosine and serum malondialdehyde and 4-hydroxyalkenals will be used as biomarkers of DNA and lipid oxidative damage, respectively, whereas uric acid as the most prominent low-molecular weight endogenous antioxidant will be measured by an automated enzymatic technique at T0, T2 and T3, to avoid possible short-term biological interferences of exercise.

Patients will provide their self-monitoring blood glucose (SMBG) records kept in accordance with their treatment algorithms and recommendations over the whole study period.

Due to a possible interaction between depression and neuropathic pain, patient self-assessments of neuropathic pain will be evaluated by a simple visual analogue scale at all points of measurement.

Indicators of glycaemic control (HbA_1c_) will be determined by an automated immunoturbidimetric method using commercially available reagents (Roche Diagnostics), traceable to the NGSP/DCCT standard, with a total imprecision (CV) of 2.8%.

Patients' preferences regarding the type of treatment (psychoeducation, physical activity intervention, treatment as usual) will be recorded. Although it is assumed that this might influence drop out, adherence and intervention impact, the methodological benefits of randomisation outweigh the potential costs. Possible effects of treatment preference on outcome measures will be controlled for statistically.

### Outcome measures

#### Primary outcome measures

The primary study outcomes will be depressive symptoms as measured by the CES-D.

#### Secondary outcome measures

Secondary outcomes will be self-management of diabetes, glycaemic control, diabetes-related distress and health-related quality of life measured as described previously.

#### Tertiary outcome measures

Tertiary outcomes will be biochemical markers reflecting common pathophysiological processes of insulin resistance, inflammation and oxidative damage that are assumed to be intertwined in both diabetes and depression.

### Data analysis

Continuous data will be summarized for each group using descriptive statistics. The distributions of continuous data at the follow-up assessments will be compared by the linear mixed-effects model the flexibility of which makes it the preferred choice for the analysis of repeated-measures data.

Categorical data will be summarized for each treatment group using counts and percentages. The distributions of categorical data in each treatment group will be compared using the Pearson Chi-square test.

In addition to the effects of intervention on psychological and biomedical outcome variables, data on participation rate among eligible individuals (intervention reach) will be considered relevant.

All analyses will be performed by StatSoft Statistica version 7.0.

### Ethical aspects

The study protocol has been approved by the Vuk Vrhovac University Clinic's Ethics Committee. Patients will be provided with information about this trial, their rights and the data to be collected from them, and a written informed consent will be obtained from all participants.

## Abbreviations

HbA_1c_: glycated haemoglobin; PHQ-2: Patient Health Questionnaire; SCID-I: Structured Clinical Interview for the DSM-IV Axis I Disorders; GP: general practitioner; CES-D: Center for Epidemiologic Studies Depression Scale; PAID: Problem Areas in Diabetes; SF-12: Short Form Survey; SDSCA: Summary of Diabetes Self-Care Activities; BMI: body mass index; WHR: waist-to-hip ratio; WBC: white blood cell count; DNA: deoxyribonucleic acid; SMBG: self-monitoring blood glucose

## Competing interests

The authors declare that they have no competing interests.

## Authors' contributions

All authors collaborated on the development of the study design. MPO is responsible for devising the interventions. MPO, DA, and MVL planned the study outcomes and their collection. MPO and DA wrote the initial draft of the manuscript. NH contributed in preparing and reviewing the manuscript.
